# Letter to editor about “Artificial intelligence-based prediction model for surgical site infection in metastatic spinal disease: a multicenter development and validation study”

**DOI:** 10.1097/JS9.0000000000003288

**Published:** 2025-08-22

**Authors:** Zhihe Zeng, Zhaoyang Xiao

**Affiliations:** Department of Anesthesiology, The Second Affiliated Hospital of Dalian Medical University, Dalian, China

*Dear Editor,*

We have carefully reviewed the recently published article in your journal entitled *“Artificial Intelligence-Based Prediction Model for Surgical Site Infection in Metastatic Spinal Disease: a Multicenter Development and Validation Study”*^[1]^. This study addresses an important clinical problem by targeting a high-risk population and employing machine learning algorithms to develop and externally validate a predictive model for postoperative SSI. Furthermore, the development of an AI-based application adds translational value and may promote precision in surgical decision-making. While we commend the authors for their efforts, we would like to offer several constructive comments regarding methodological design, variable handling, clinical relevance of the model, and comparison with existing tools. The present study was conducted and reported following the TITAN guideline^[2]^.

First, the authors point out that the incidence of SSI following surgery for metastatic spinal disease is higher than in conventional spinal procedures (6.4–7.7%), likely due to factors such as tumor-related immunosuppression, surgical complexity, and patient comorbidities. This context is clinically meaningful. However, the definition of the model’s intended application scenario remains relatively broad, and the study does not provide a systematic comparison with established risk scoring tools such as *SpineSage*^[3]^. Moreover, although the model achieved an AUC of 0.986 in the training cohort, its performance declined notably in external validation (AUC 0.84), suggesting possible overfitting or issues related to heterogeneity between cohorts. The lack of direct comparisons with traditional scoring systems – using metrics such as Net Reclassification Improvement (NRI), Integrated Discrimination Improvement (IDI), or net clinical benefit – makes it difficult to evaluate the added value of this model within clinical workflows.

Second, the handling of variables could be more transparent. The study states that only preoperative and intraoperative features were used to enable early prediction. However, the full list of variables and their preprocessing (e.g., strategies for handling missing data and outliers) were not detailed in the main text. It is also unclear whether closely related intraoperative variables – such as blood loss and transfusion requirements – were included or excluded. This lack of clarity limits the model’s reproducibility and external applicability. Additionally, the study did not incorporate multilevel modeling approaches (e.g., hierarchical logistic regression or nested cross-validation), which may constrain the model’s ability to capture complex interactions or nonlinear effects. Even though a Gradient Boosting Machine (GBM) was used, further assessment of variable importance consistency and stability would strengthen the model’s credibility.

Third, although the authors reviewed several SSI prediction models across different surgical fields – including third molar extraction, pelvic floor surgery, and oncological resections^[4,5]^ – and acknowledged an existing model for spinal metastasis wound complications (AUC 0.81)^[6]^, the comparisons were mostly limited to surface-level AUC metrics. A more in-depth discussion of key differences in algorithm selection (e.g., random forest vs. XGBoost vs. deep learning), types of variables (e.g., inclusion of postoperative time-series or biomarker data), and data sources would have better positioned this study within the current research landscape. Regarding the SSI definition, the authors adopted a 30-day follow-up window per CDC guidelines. While this is standard, implant-associated infections – especially deep or organ-space infections – can often manifest after 90 days or more^[6]^. Therefore, the study may underestimate the true SSI incidence. Future research should consider extended follow-up periods and stratify infections by depth (superficial, deep, or organ-space) to enhance the model’s clinical interpretability.

In conclusion, this study represents an important step forward in the development and potential application of predictive models for SSI in spinal oncology. To further improve its generalizability and facilitate real-world adoption across diverse populations and clinical settings, we recommend addressing the above considerations in future work.

## Data Availability

Not applicable.
